# A 21 day Daniel Fast improves selected biomarkers of antioxidant status and oxidative stress in men and women

**DOI:** 10.1186/1743-7075-8-17

**Published:** 2011-03-18

**Authors:** Richard J Bloomer, Mohammad M Kabir, John F Trepanowski, Robert E Canale, Tyler M Farney

**Affiliations:** 1 Cardiorespiratory/Metabolic Laboratory The University of Memphis Memphis, TN 38152, USA

## Abstract

**Background:**

Dietary modification via both caloric and nutrient restriction is associated with multiple health benefits, some of which are related to an improvement in antioxidant status and a decrease in the production of reactive oxygen species. The Daniel Fast is based on the Biblical book of Daniel, is commonly partaken for 21 days, and involves food intake in accordance with a stringent vegan diet. The purpose of the present study was to determine the effect of a 21 day Daniel Fast on biomarkers of antioxidant status and oxidative stress.

**Methods:**

43 subjects (13 men; 30 women; 35 ± 1 yrs; range: 20-62 yrs) completed a 21 day Daniel Fast following the guidelines provided by investigators. Subjects reported to the lab in a 12 hour post-absorptive state both pre fast (day 1) and post fast (day 22). At each visit, blood was collected for determination of malondialdehyde (MDA), hydrogen peroxide (H_2_O_2_), nitrate/nitrite (NOx), Trolox Equivalent Antioxidant Capacity (TEAC), and Oxygen Radical Absorbance Capacity (ORAC). Subjects recorded dietary intake during the 7 day period immediately prior to the fast and during the final 7 days of the fast.

**Results:**

A decrease was noted in MDA (0.66 ± 0.0.03 vs. 0.56 ± 0.02 μmol L^-1^; p = 0.004), while H_2_O_2 _demonstrated a trend for lowering (4.42 ± 0.32 vs. 3.78 ± 0.21 μmol L^-1^; p = 0.074). Both NOx (18.79 ± 1.92 vs. 26.97 ± 2.40 μmol L^-1^; p = 0.003) and TEAC (0.47 ± 0.01 vs. 0.51 ± 0.01 mmol L^-1^; p = 0.001) increased from pre to post fast, while ORAC was unchanged (5243 ± 103 vs. 5249 ± 183 μmol L^-1 ^TE; p = 0.974). As expected, multiple differences in dietary intake were noted (p < 0.05), including a reduction in total calorie intake (2185 ± 94 vs. 1722 ± 85).

**Conclusion:**

Modification of dietary intake in accordance with the Daniel Fast is associated with an improvement in selected biomarkers of antioxidant status and oxidative stress, including metabolites of nitric oxide (i.e., NOx).

## Background

Reducing daily caloric intake has been reported to improve overall health in both humans [1] and animals [2], and to increase lifespan in a variety of species [3]. While many hypotheses have been put forth in an attempt to explain these findings, two in particular have received the greatest amount of attention: the hormesis hypothesis and the attenuation of oxidative damage hypothesis. The former proposes that caloric restriction, by acting as a low-intensity stressor, increases an organism's stress tolerance in an attempt to improve health and survival [4]. The latter proposes that caloric restriction improves health and extends life by attenuating oxidative stress [5], a finding that is well-documented in the literature [6].

Oxidative stress is brought about by an increase in the production of reactive oxygen species (ROS) and/or a decrease in antioxidant defense [7]. This phenomenon can potentially result in oxidative damage to nucleic acids, lipids, and proteins, which may contribute over time to the development of human disease [8], as well as to the aging process. The elevation in ROS production appears to be influenced not merely by the amount of dietary energy provided by a given meal [9] but also by the macronutrient composition of the meal [9-12]. For example, we have recently shown that the metabolism of dietary lipid (in the form of saturated fat) results in a greater increase in oxidative stress biomarkers than the metabolism of either dietary protein or carbohydrate [10]. Other work corroborates the significant increase in oxidative stress biomarkers that we observed, following ingestion of saturated fat [13-15] and simple carbohydrate [16-18]. In comparison, minimal increases in oxidative stress have been noted following ingestion of monounsaturated fatty acids [19] and complex carbohydrates [20].

It has been noted that foods containing little to no saturated fat, along with an abundance of fiber and micronutrients (e.g. fruits, vegetables, whole grains, nuts and seeds), provide health-enhancing properties [21-23]. Clearly, a diet consisting of the above foods is rich in antioxidant micronutrients [24,25] and is associated with improved blood antioxidant status [26,27]. Moreover, such a diet has been shown to result in a lowering of oxidative stress biomarkers [28-30]. Finally, diets rich in polyphenols are known to favorably impact endothelial function [31], which may be explained by an increase in nitric oxide bioavailability. The increase in nitrate intake associated with plant-based diets may also explain improvements in endothelial function [32]. The measurement of total nitrates and nitrites is often used as a surrogate marker for nitric oxide, a molecule with multiple physiological functions [33,34], particularly those related to vaso-relaxation [35]. Collectively, the physiological benefits mentioned in this paragraph may be at least partly responsible for the findings of improved cardiovascular [36] and metabolic [37] health observed in individuals consuming diets that are abundant in such "natural" foods.

The Daniel Fast involves *ad libitum *intake of fruits, vegetables, whole grains, nuts, seeds, and oil. This plan resembles a vegan diet, which has been reported to yield health-enhancing properties [38-40]. However, a Daniel Fast is more stringent due to the fact that, in addition to the prohibition of animal products, intake of the following is disallowed: processed foods, white flour products, preservatives, additives, sweeteners, flavorings, caffeine, and alcohol. These additional restrictions may be associated with more robust findings pertaining to improved health-related outcomes than those associated with vegan diets.

The concept of the Daniel Fast comes from two passages within Biblical text, Daniel 1:8-14 and Daniel 10:2-3. In both passages, the Lord's servant Daniel "ate no rich-food or meat and drank no wine", as first reported in Daniel 1:8-14 (for a period of 10 days) and then in Daniel 10:2-3 (for a period of 21 days). Because the latter passage describes a classical fast to coincide with a period of mourning, a modern day Daniel Fast is most commonly followed for 21 days [41]. This fast continues to increase in popularity, as evidenced by two recently published books on the topic [42,43], and over 6 million Google™ hits noted on 2/15/11 using the term "Daniel Fast".

The purpose of the present study was to investigate the effects of a 21 day Daniel Fast on biomarkers of oxidative stress, antioxidant status, and nitric oxide in human subjects. To our knowledge, this was the first scientific investigation of the Daniel Fast in relation to markers of oxidative stress. As such, this study simply involved a pre/post assessment and did not include random assignment to two different dietary plans.

## Methods

### Subjects and Screening

Forty-three subjects (13 men; 30 women; mean age = 35 ± 1 years) were enrolled in this study. Six female subjects were vegetarians. One female subject had a diagnosis of well-controlled type II diabetes and one male subject had a history of coronary artery bypass graft surgery. Three subjects had elevated blood pressure upon screening (BP ≥ 140/90 mmHg; 2 men and 1 woman) and seven subjects had elevated total cholesterol (> 200 mg dL^-1^; 1 man and 6 women). Subject characteristics are presented in Table 1 and medication use of subjects is presented in Table 2.

**Table 1:** Subject baseline characteristics

Variable	Value
Heart Rate (bpm)	68.2 ± 1.7
Systolic Blood Pressure (mmHg)	114.7 ± 2.3
Diastolic Blood Pressure (mmHg)	72.2 ± 1.6
Weight (kg)	77.5 ± 3.0
BMI (kg m^-2^)	27.0 ± 0.9
Waist (cm)	92.2 ± 2.0
Hip (cm)	105.8 ± 1.8
Waist:Hip ratio	0.87 ± 0.01
Total Body Fat (%)†	30.2 ± 1.6
Fat Mass (kg)	24.2 ± 1.9
Fat Free Mass (kg)	53.8 ± 2.0

Values are mean ± SEM.

† Assessed using dual energy x-ray absorptiometry (DEXA).

**Table 2:** Medication usage of subjects

Medication	Number of Subjects
Sulfonylurea	1 (woman)
Statin	1 (man); 1 (woman)
Plavix^®^	1 (man)
Beta blocker	1 (man)
Angiotensin II receptor antagonist	1 (woman)
Anti-depressant	1 (man); 3 (women)
Sleep aid	2 (women)
Oral contraceptives	6 (women)
Hormone replacement therapy	2 (women)

No restrictions were placed on subjects regarding body mass necessary for enrollment. Hence, the BMI of subjects ranged from 18.0 kg m^-2 ^to 40.6 kg m^-2^; with 21 subjects classified as normal weight (BMI < 25 kg m^-2^), 9 classified as overweight (BMI 25-29.9 kg m^-2^), and 13 classified as obese (BMI ≥ 30 kg m^-2^). All subjects were nonsmokers. Thirty-four subjects were classified as exercise-trained, performing 1.8 ± 0.24 hours of anaerobic and 3.0 ± 0.23 hours of aerobic exercise per week for the past 5.4 ± 0.82 and 7.7 ± 1.0 years, respectively. Based on the above information and aside from the overweight or obese status of 22 subjects, most were considered "relatively" healthy and active. Medical and activity data were obtained via physical assessment and questionnaires. Each subject was informed of all procedures, potential risks, and benefits associated with the study through both verbal and written form in accordance with the approved procedures of the University Institutional Review Board for Human Subjects Research (H10-06). All subjects signed an informed consent form prior to participating.

During the initial visit to the laboratory, subjects completed all paperwork and had hemodynamic and anthropometric variables measured. Subjects were provided detailed instructions for the fast and were given food logs for dietary recording. Subjects were provided an outline of foods that are allowed on the fast as well as commonly consumed foods that are disallowed. A basic recipe guide was also provided. It is important to note that subjects purchased and prepared their own food during the entire fast. The investigators simply provided initial instruction and guidance when needed. In contrast to studies in which subjects are provided with all meals in an attempt to enhance compliance, our method placed the responsibility of food purchase, preparation, and intake directly on the subject. We believe that this approach has greater practical application with regards to a dietary intervention. Following this initial screening visit, subjects returned to the lab 1-2 weeks later to have baseline assessments performed and to begin the 21 day fast. Outcome variables were measured before (baseline: day 1 of the fast) and after the fast (day 22). All data collection was done in the morning hours (5:00-11:00am) and with subjects in a 12 hour post-absorptive state.

### Blood Collection and Biochemical Variables

Venous blood samples were taken from subjects' forearm via needle and Vacutainer™ tubes by a trained phlebotomist. Following collection, blood collected in tubes containing ethylenediaminetetraacetic acid (EDTA) was immediately separated to plasma by centrifugation at 1500g for 15 minutes at 4°C. Blood collected in tubes containing no additives was allowed to clot at room temperature for 30 minutes and then separated to serum by centrifugation at 1500g for 15 minutes at 4°C. Plasma and serum samples were immediately stored in multiple aliquots at -70°C until analyzed. All blood samples were assayed for malondialdehyde (MDA), hydrogen peroxide (H_2_O_2_), nitrate/nitrite (NOx), Trolox Equivalent Antioxidant Capacity (TEAC), and Oxygen Radical Absorbance Capacity (ORAC).

Malondialdehyde was analyzed in plasma following the procedures of Jentzsch et al. [44] using reagents purchased from Northwest Life Science Specialties (Vancouver, WA). Specifically, 75 μL of plasma was added to microcentrifuge reaction tubes with the addition of 3 μL of butylated hydroxytoluene in methanol to minimize *ex vivo *lipid peroxidation. 75 μL of 1M phosphoric acid and 75 μL of 2-thiobarbituric acid reagent were added to each reaction tube and mixed thoroughly. Samples and reagents were incubated for 60 minutes at 60°C. Following incubation, the reaction mixture was transferred to a microplate, and the absorbance was read using a spectrophotometer at both 535 and 572nm to correct for baseline absorption. Malondialdehyde equivalents were calculated using the difference in absorption at the two wavelengths. Quantification was performed with a calibration curve using tetramethoxypropane in a stabilizing buffer. The coefficient of variation (CV) for this assay in our lab is < 8%.

Hydrogen peroxide was analyzed in plasma using the Amplex Red reagent method as described by the manufacturer (Molecular Probes, Invitrogen Detection Technologies, Eugene, OR). In the reaction mixture, H_2_O_2_, in the presence of horseradish peroxidase, reacts with Amplex Red reagent to generate the red-fluorescence oxidation product, resorufin. Quantification was performed with a calibration curve. The CV for this assay in our lab is < 8%.

Nitrate/nitrite was analyzed in plasma using a commercially available colorimetric assay kit (Caymen Chemical, Ann Arbor, MI) according to the procedures provided by the manufacturer. Thawed plasma samples were centrifuged at 10,000g for 5 minutes in a refrigerated centrifuge (4°C). Following the addition of nitrate reductase co-factor to each diluted sample, nitrate reductase was added and the mixture was incubated for three hours to allow for the full conversion of nitrate to nitrite. Greiss reagent was then added, which converts nitrite into a deep purple azo compound. The absorbance was then detected photometrically at 540 nm. Quantification was performed with a calibration curve. The CV for this assay in our lab is < 8%.

Antioxidant capacity was analyzed in serum via the TEAC assay using procedures outlined by the reagent provider (Sigma Chemical, St. Louis, MO). Quantification was performed with a calibration curve. The CV for this assay in our lab is < 4%. Antioxidant capacity was also analyzed in serum (following a 750 fold dilution) via the Oxygen Radical Absorbance Capacity (ORAC) assay using procedures outlined by the reagent provider (Zen-Bio, Inc.; Research Triangle Park, NC). The CV for this assay in our lab is < 4%. It should be noted that several methods are available to assess the **"**total" antioxidant capacity of blood. These include the TEAC assay (which appears to be primarily influenced by urate), the ORAC assay, the ferric-reducing ability of plasma (FRAP) assay, and the total radical-trapping antioxidant parameter (TRAP) assay. Of these, ORAC and FRAP have been noted to be well-correlated, while TEAC has little correlation with either ORAC or FRAP [45]. Therefore, we chose to include more than one antioxidant capacity marker, which has been suggested previously [46].

### Dietary Records and Physical Activity

All subjects were instructed to maintain their normal diet until they began the fast. During the seven days immediately prior to the fast, subjects recorded all food and beverage intake. Subjects were also asked to record food and beverage intake during the final seven days of the fast. Records were reviewed with each subject for accuracy and then analyzed using Food Processor SQL, version 9.9 (ESHA Research, Salem, OR). Regarding physical activity, subjects were instructed to maintain their normal habits during the entire study period with one notable exception: subjects were instructed not to perform strenuous exercise during the 48 hours immediately preceding the two assessment days. Subjects were also instructed to refrain from alcohol consumption during the fast, as well as during the 48 hours that preceded day 1.

### Compliance and Additional Variables

On a scale of 0-100 (0 = complete non-compliance, 100 = complete compliance), subjects were asked to rate their overall compliance to the fast with regards to food choices. Pre and post assessment data aside from those indicated within the present manuscript - including anthropometric, blood lipid, and other hematological measures - are presented elsewhere [41].

### Statistical Analysis

Biochemical data were analyzed using a paired t-test. In addition, sex, exercise training status (trained or untrained), and weight status (normal weight, overweight, or obese) were used in an analysis of variance (ANOVA) to determine the influence of these variables on the changes in our biomarkers from pre to post fast. Analyses were performed using JMP statistical software (version 4.0.3, SAS Institute, Cary, NC). Statistical significance was set at P ≤ 0.05. The data are presented as mean ± SEM.

## Results

### Compliance

The overall response of subjects to the fast was positive. Subjects noted that they enjoyed the *ad libitum *nature of the fast as well as the wide variety of food choices. Most subjects reported that they would continue implementing many components of the eating plan into their usual diets. Self-rated compliance to the fast was reported to be 98.7 ± 0.2%.

### Anthropometric Data

Although previously presented elsewhere [41], it should be noted that body weight was reduced by an average of 2.8kg from pre to post fast, with fat mass accounting for approximately 40% of the reduction. Waist and hip circumference measures were decreased by approximately 1.5cm from pre to post fast.

### Dietary Data

As expected, several differences existed in dietary intake from pre to post fast. These included a decrease in total calories (p = 0.0005), protein grams (p = 0.0003), percentage of protein intake (p = 0.004), fat grams (p = 0.003), saturated fat (p < 0.0001), trans fat (p = 0.006), and cholesterol (p < 0.0001). An increase in the percentage of carbohydrate intake (p = 0.0002), fiber (p < 0.0001), and vitamin C (p = 0.002) was also noted. Data are presented in Table 3.

**Table 3:** Dietary data of men and women before and during the final seven days of a 21 day Daniel Fast

Variable	Pre	During	P value
Calories	2185 ± 94	1722 ± 85	0.0005
Protein (g)	92 ± 6	62 ± 5	0.0003
Protein (%)	17 ± 0	13 ± 0	0.004
Carbohydrate (g)	287 ± 14	269 ± 17	0.41
Carbohydrate (%)	53 ± 0	62 ± 0	0.0002
Fiber (g)	26 ± 2	40 ± 3	< 0.0001
Sugar (g)	95 ± 7	86 ± 6	0.37
Fat (g)	74 ± 5	54 ± 4	0.003
Fat (%)	30 ± 0	27 ± 0	0.20
Saturated Fat (g)	24 ± 2	9 ± 1	< 0.0001
Monounsaturated Fat (g)	14 ± 2	14 ± 2	0.89
Polyunsaturated Fat (g)	8 ± 1	9 ± 1	0.47
Trans Fat (g)	1 ± 0	0 ± 0	0.006
Omega 3 (mg)	711 ± 163	798 ± 202	0.77
Omega 6 (mg)	2510 ± 327	3341 ± 345	0.10
Cholesterol (mg)	225 ± 19	28 ± 20	< 0.0001
Vitamin C (mg)	70 ± 9	119 ± 12	0.002
Vitamin E (mg)	8 ± 2	11 ± 1	0.36
Vitamin A (RE)	404 ± 60	435 ± 70	0.70

Values are mean ± SEM.

### Biochemical Data

Related to the bloodborne variables, we were able to obtain blood samples from 42 of the 43 subjects. Therefore, bloodborne data presented within are specific to a sample of 42 subjects (13 men and 29 women). With regards to the ANOVA, no interactions were noted for any measured variable (p > 0.05). However, a sex main effect was noted for MDA (p = 0.01), with lower values for women (0.57 ± 0.02 μmol L^-1^) as compared to men (0.67 ± 0.03 μmol L^-1^). An exercise training status main effect was noted for TEAC (p = 0.03), with lower values for trained subjects (0.48 ± 0.01 mmol L^-1^) as compared to untrained subjects (0.54 ± 0.02 mmol L^-1^). A trend was noted for weight status with regards to H_2_O_2 _(p = 0.07), with higher values for obese subjects (4.7 ± 0.4 μmol L^-1^) as compared to overweight (3.6 ± 0.4 μmol L^-1^) and normal weight (3.8 ± 0.3 μmol L^-1^) subjects. No other main effects were noted (p > 0.05).

**Figure 1:**
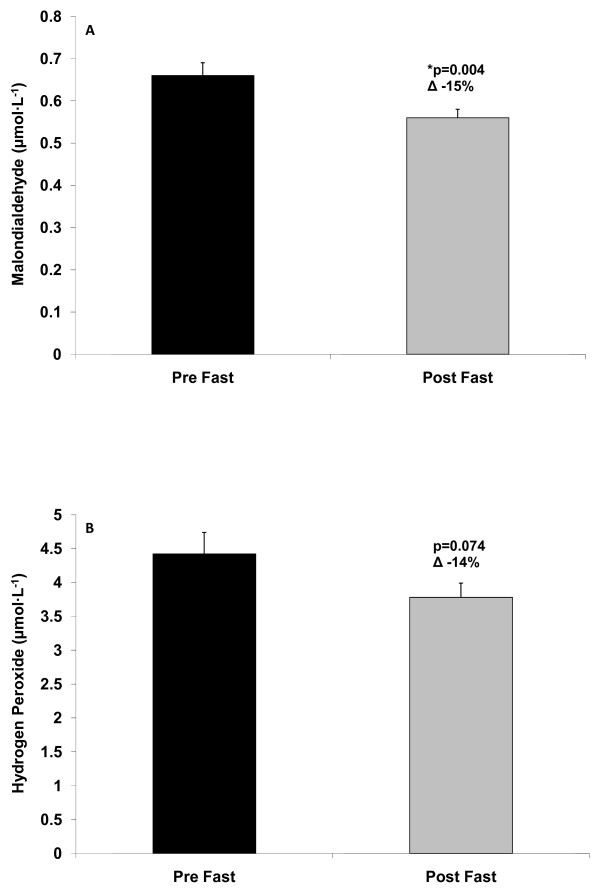
**Plasma malondialdehyde (A) and plasma hydrogen peroxide (B) of men and women before and after a 21 day Daniel Fast**. Values are mean ± SEM.

**Figure 2:**
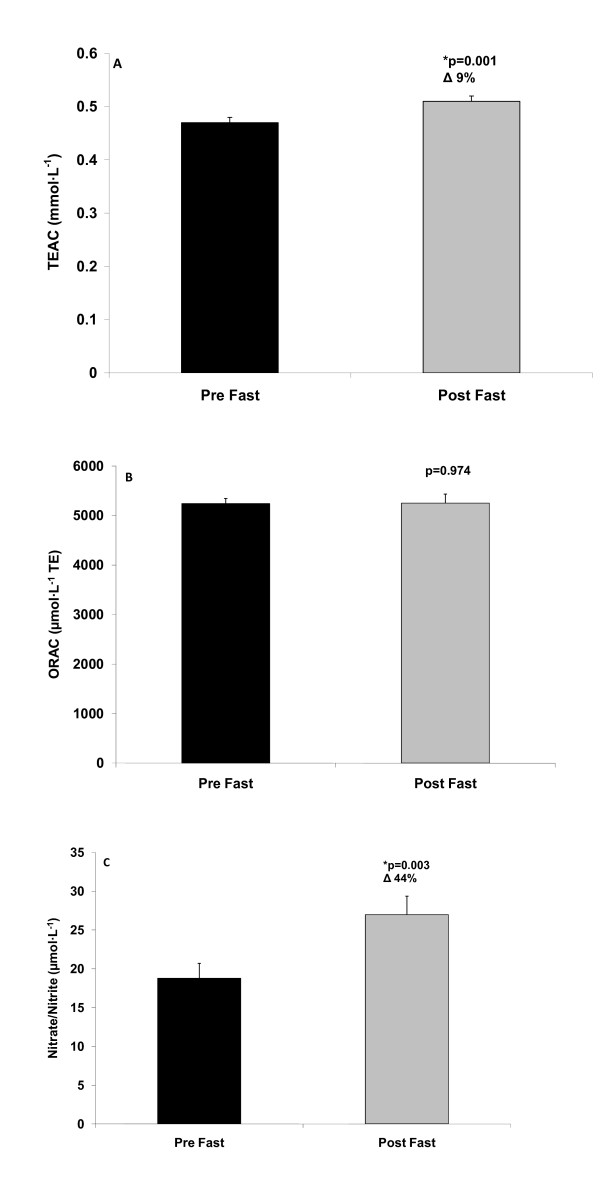
**Serum Trolox Equivalent Antioxidant Capacity (A), serum Oxygen Radical Absorbance Capacity (B), and plasma nitrate/nitrite (C) of men and women before and after a 21 day Daniel Fast**. Values are mean ± SEM.

With regards to the paired t-tests, the results were as follows: Significant decreases were noted in MDA (p = 0.004; Figure 1A). H_2_O_2 _was lower post fast compared to pre fast, but this reduction did not reach statistical significance (p = 0.074; Figure 1B). TEAC was increased significantly from pre to post fast (p = 0.001; Figure 2A), ORAC was unchanged (p = 0.974; Figure 2B), and NOx was increased significantly from pre to post fast (p = 0.003; Figure 2C).

## Discussion

Results from the present study indicate that a 21 day Daniel Fast increases NOx and improves selected measures of antioxidant status and oxidative stress. The fast was well-tolerated, as evidenced by a self-rated compliance of 98.7 ± 0.2%, suggesting that longer-term maintenance of such a dietary plan may be feasible. To our knowledge, this is the first scientific investigation to assess the impact of the Daniel Fast on biomarkers of oxidative stress. Moreover, this study extends the findings of other plant-based diets by documenting the impact of a strict vegan diet on multiple measures of oxidative stress and antioxidant capacity.

It is important to note that our findings are in reference to relatively healthy, young to middle age men and women (age range: 20-62 years), with a wide BMI range (18.0 kg m^-2 ^to 40.6 kg m^-2^), and varied exercise and dietary habits. Interestingly, we noted similar findings across subject groups when comparing data from pre to post fast. However, while the overall response to the fast was not different between subject groups (i.e., men vs. women, exercise- trained vs. untrained, obese vs. overweight vs. normal weight), it should be noted that some overall differences across subject groups did occur with regards to MDA and TEAC, and to a lesser extent H_2_O_2_. For example, MDA was lower for women (0.57 ± 0.02 μmol L^-1^) as compared to men (0.67 ± 0.03 μmol L^-1^) and TEAC was lower for trained subjects (0.48 ± 0.01 mmol L^-1^) as compared to untrained subjects (0.54 ± 0.02 mmol L^-1^). Additionally, H_2_O_2 _was slightly but insignificantly (p = 0.07) higher for obese subjects (4.7 ± 0.4 μmol L^-1^) as compared to overweight (3.6 ± 0.4 μmol L^-1^) and normal weight (3.8 ± 0.3 μmol L^-1^) subjects. These findings are supported by previous literature, in particular with regards to higher oxidative stress in men compared to women [47] and in obese compared to overweight and/or normal weight subjects [48,49]. With regards to the antioxidant capacity in exercise-trained compared to untrained subjects, mixed results are available; some supporting our finding of lower antioxidant capacity in trained individuals [50] and some refuting this [51,52]. Those findings in support of our data may be linked to the acute increase in ROS experienced by exercise-trained individuals, which may partially deplete endogenous antioxidants over time [50]. Of course, in accordance with the principle of hormesis [53], the opposite effect may occur as a result of acute elevations in ROS. This is an area of investigation that requires further attention and is likely dependent on the volume and intensity of the exercise sessions, coupled with post exercise recovery strategies.

In as few as three weeks, we noted a 15% reduction in MDA, a lipid-specific marker of oxidative stress. This reduction is greater than those noted in studies incorporating other plant-based diets [29,54], which may be due to the omission of many other food components (e.g., additives and preservatives) in the current Daniel Fast plan. A similar percent decrease was noted for H_2_O_2 _(14%), a variable that we have not seen reported in reference to other plant-based diets. Collectively, these findings indicate that a 21 day Daniel Fast can reduce blood oxidative stress biomarkers, which may potentially be associated with improved health [55] and increased longevity [56]. These findings are supported by a 9% increase in antioxidant capacity, as measured by TEAC; although ORAC values were unchanged. Regarding the discrepancy in the TEAC and ORAC findings, it has been noted that these two measures are not well correlated [45] despite the fact that both provide an indication of antioxidant capacity.

Interestingly, many subjects did experience an increase in ORAC from pre to post fast; however, some subjects experiencing a significant decrease in ORAC also decreased caloric intake considerably (~50%), potentially masking any group increase in this marker. When investigating changes in other biomarkers for these subjects from pre to post fast, they followed the overall findings of the group (e.g., decrease in MDA and increase in NOx). Therefore, while a decrease in caloric intake of this magnitude (~50%) may not be considered optimal for purposes of general health, our collective findings indicate that such a change may not be problematic for purposes of improving the oxidative stress profile (with the possible exception of blood ORAC). Further study pertaining to the effects of a Daniel Fast on serum TEAC, ORAC, and other antioxidant and oxidative stress biomarkers is needed.

Finally, NOx increased 44% from pre to post fast, which may be associated with greater nitric oxide bioavailability and decreased production of peroxynitrite [57]. The increase in NOx in this investigation, which may be a result of increased vegetable intake [32] and/or decreased ROS production [57], may enhance blood flow due to nitric oxide's important role in endothelial dependent vaso-relaxation [35]. Nitric oxide is well-recognized as an important signaling molecule involved in numerous biological processes including, but not limited to, smooth muscle relaxation, attenuation of smooth muscle cell proliferation, inhibition of platelet and leukocyte aggregation, and immune defense [33,34]. As such, it is possible that an increase in NOx may be associated with improved cardiovascular health [58].

It is interesting to note that the changes highlighted above were observed in a sample of subjects who, for the most part, were considered to be relatively healthy at the onset of the fast. While many subjects were classified as overweight (n = 9) or obese (n = 13) based on BMI, most were normal weight (n = 21), and 34 of the 43 subjects exercised regularly. Regardless of weight classification, all subject groups demonstrated improvements in the measured variables (as indicated in the Results section). Considering that our subjects may have had less "room for improvement" as compared to many individuals beginning a new dietary program, it is possible that we may have observed more robust effects if we had recruited a homogenous sample of individuals who were classified as unhealthy to begin with (e.g., sedentary, obese, known metabolic or cardiovascular disease).

Also of interest, we noted similar mean changes between vegetarians and omnivores in our sampled biomarkers. For example, the six vegetarian subjects in our study experienced a 10% reduction in MDA and a 79% increase in NOx from pre to post fast. These results suggest that the exclusion of meat from the diet is not the only dietary factor responsible for altering oxidative stress, antioxidant capacity, or nitric oxide metabolites. It is likely that multiple dietary factors - particularly the exclusion of additives, preservatives, and high-glycemic, processed carbohydrate foods, in addition to the focused inclusion of fresh and frozen fruits and vegetables - positively affected the biochemical variables measured in the present design.

Related to the above, while it is obvious that the change in subjects' dietary intake is responsible for our findings, we are uncertain as to which specific variables had the greatest impact. Because we have included multiple variables in this study, in addition to the measurement of several dietary variables, a complete analysis of predictor and response variables deserves attention. As we have recently completed an additional study of the Daniel Fast, greatly increasing the needed sample size for multiple regression analysis of this scope, this is now the focus of a separate manuscript. Because variables such as body mass, sex, exercise training status, and dietary variables, as well as changes in certain anthropometric and biochemical variables (e.g., blood lipids) may all influence our measured outcome variables, all may be considered in such an analysis--to determine which factors are most influential in predicting the change in our measured outcomes. With only a cursory view, we believe that the combination of decreased calorie and saturated fat intake, together with an increase in nutrient- and fiber-rich fruit, vegetable, and whole grain intake, contributed to our findings. It is also possible that the elimination of food additives, preservatives, and processing agents, in addition to the decrease in protein intake (methionine included [59]), could be responsible for our findings.

Considering our collective results, it is important to point out some limitations of this work. First, while it is possible that our data may have clinical implications, particularly for individuals prone to oxidative stress-related ill-health and disease, we cannot state this with confidence. The majority of our subjects did not have known disease and actually had relatively low levels of oxidative stress biomarkers at the start of the fast. Although elevated oxidative stress appears related to disease progression [8], it is unknown what impact a slight reduction in oxidative stress would have on relatively healthy individuals who do not have elevated oxidative stress to begin with. Further work with the inclusion of subjects with known cardiovascular and metabolic disease, in particular those with elevated oxidative stress biomarkers, would be needed to determine the clinical relevance of these potential changes. This could be done with the inclusion of healthy subjects within the design, to allow for the comparison of this fast between those with known disease and those who are without disease. It is possible that the findings may differ between the two groups. Second, because our protocol merely followed subjects over a 21 day period, long-term compliance to this eating plan needs to be determined. While it is possible that more favorable results may be observed with a longer intervention period, subjects would first need to have success in compliance for such results to be realized. Third and related to the above, disease progression involves multiple components and typically follows an extended time course. Therefore, future studies should be longer in length- possibly several months in duration- in order to more completely determine the effects of this eating plan on biomarkers of human health. Fourth, while we included biochemical markers of oxidative stress in the current design, data pertaining to functional outcomes of health (e.g., endothelial function, physical performance) are needed to extend these findings. Changes in the design to address the above limitations may allow for more insight to be gained pertaining to both the practical and clinical relevance of the Daniel Fast eating plan.

## Conclusion

Our data indicate that a 21 day Daniel Fast decreases blood oxidative stress, increases antioxidant capacity (as measured by TEAC), and increases NOx. Although further work is needed to extend these initial findings, our data provide important insight into the ability of this Biblically-based fast to improve biomarkers of human health.

## Competing interests

The authors declare that they have no competing interests.

## Authors' contributions

RJB was responsible for the study design, oversight and/or analysis of biochemical variables, statistical analyses, and writing of the manuscript. MMK was responsible for coordination of the study. MMK, JFT, REC, and TMF were responsible for subject recruitment, screening and retention, data collection and entry, and blood collection and processing. All authors assisted in reviewing/editing the manuscript and all authors reviewed and approved of the final manuscript.
